# Correlation Between 1p19q Status and Various Biomarkers in Selected Central Nervous System Tumors

**DOI:** 10.7759/cureus.108215

**Published:** 2026-05-04

**Authors:** Ezgi Akar, Ugur Ture, Murat Aydin Sav

**Affiliations:** 1 Neurosurgery, Haydarpaşa Numune Training and Research Hospital, Istanbul, TUR; 2 Neurosurgery, Yeditepe University, Istanbul, TUR; 3 Pathology, Yeditepe University, Istanbul, TUR

**Keywords:** 1p19q codeletion, biomarker, co-deletion, glial tumor, mgmt

## Abstract

Background and objective: H mutations and 1p19q co-deletion are the main biomarkers used in the nomenclature of glial tumors. Detection of 1p19q co-deletion directly diagnoses oligodendroglioma. The primary objective of this study was to evaluate the 1p19q co-deletion status in diffuse gliomas in accordance with the World Health Organization (WHO) integrated classification framework. In addition, we aimed to investigate the presence of 1p19q alterations in other, less frequently encountered glial-derived central nervous system tumors.

Methods: We retrospectively analyzed the biomarker findings obtained from the pathology preparations of 167 cases of glial brain tumors (93M, 74F) operated in the Department of Neurosurgery at Yeditepe University Hospital and whose preparations were investigated in the pathology laboratory between 2019 and 2021. The data was statistically analyzed.

Results: ATRX, IDH1, EGFR, GFAP, and OLIG2 positivity were associated with the positivity of 1p19q co-deletion. Our study showed that only 4% of tumors with no O6-methylguanine-DNA methyltransferase (MGMT) methylation showed 1p19q co-deletion positivity. Tumors with low (0-20%) and moderate (21-60%) levels of MGMT methylation showed higher rates of 1p19q co-deletion positivity. However, tumors with high (81-99%) and complete (100%) MGMT methylation had lower rates of 1p19q co-deletion positivity.

Conclusions: The detection of 1p19q co-deletion in other tumors and its peculiar relationship with MGMT methylation made us think of the complexity of tumor biology and how both genetic and epigenetic factors interplay to influence tumor behavior and response to therapy.

## Introduction

Glial tumors, which constitute 80% of malignant brain tumors, are grouped as well-differentiated (low grade, grade 2) or undifferentiated (anaplastic, grades 3-4) according to their behavior [1]. Most gliomas are astrocytomas (approximately 70% of all brain tumors), followed by oligodendrogliomas (10-30%) [1,2]. Grade 1 glial tumors (pilocytic astrocytoma) constitute 5-6% of all glial tumors and occur primarily in children and young adults. World Health Organization (WHO) grade 2 tumors constitute 10-15% of all diffuse glial tumors, have a differentiated structure, and are most common at the age of 30-40 years. Glioblastomas are the most common type of glial tumor that develops in the brain, accounting for 60-75% of all astrocytic-glial brain tumors [3].

According to the WHO 2016 diffuse glial tumor classification, detecting 1p19q co-deletion in gliomas with IDH1 and/or IDH2 mutation is diagnostic for oligodendrogliomas [4,5]. 1p19q co-deletion is a known genetic marker of oligodendrogliomas and is associated with increased survival and sensitivity to chemo-radiotherapy.

Although 1p19q co-deletion is diagnostic for oligodendroglioma, it can also be present in other glial tumors, especially glioblastoma. The 1p19q co-deletion rates are 70.8% in grade 3 oligodendrogliomas and 23.1% in grade 3 astrocytomas [3,6,7].

DNA methylation abnormalities are associated with good prognosis and response to chemotherapy in gliomas [6]. O6-methylguanine-DNA methyltransferase (MGMT) mRNA and protein levels are associated with resistance to alkylating agents. MGMT promoter methylation is a marker of response to temozolomide treatment in grade 3 and grade 4 gliomas and, thus, a marker of good prognosis [6]. MGMT promoter methylation is low, especially in patients with glioblastomas over 65-70 years of age, and response to temozolomide was low in these patients [8].

By identifying many genes and genetic alterations related to tumorigenesis in diffuse glial tumors, the behavior and treatment of these disorders, and perhaps prevention in the long term, will be possible. There are many studies on 1p19q, a known genetic abnormality for oligodendrogliomas [4]. Our study aims to investigate the relationship with other biomarkers in diffuse and other glial tumors with and without 1p19q co-deletion.

## Materials and methods

Patient population

We retrospectively analyzed the biomarker findings obtained from the pathology preparations of 167 cases of glial brain tumors (93M, 74F) operated in the Department of Neurosurgery at Yeditepe University Hospital and whose preparations were evaluated in the pathology laboratory between 2019 and 2021. All cases were diagnosed as glial tumors of varying degrees using the WHO 2016 classification since they were operated on in 2021 and before. Patients did not receive preoperative chemo-radiotherapy. The biomarker panel results and selected fluorescence in situ hybridization (FISH) findings were analyzed. All data were extracted from pathology reports, including ATRX, IDH, GFAP, EGFR, OLIG, MGMT, H3K27M, CD34, Ki67, P53, SYN, NEU-N, NFP, PD-L1, WT-1, VIMENTIN, EMA, PTERT, P16, YAP-1, GAB-1, and 1p19q.

Histopathological and immunohistochemical evaluation

Tumor tissue samples were fixed with formalin and then embedded in paraffin blocks. Tissue sections 5-micron-thick were taken. Each slide was stained with hematoxylin-eosin (H&E). After histological evaluation, tumor-rich areas were identified on H&E-stained slides, and DNA was extracted from the corresponding tumor tissue sections. TERT promoter mutation analysis was performed by polymerase chain reaction (PCR) amplification followed by Sanger sequencing. Sequencing targeted the C228T and C250T hotspot mutations in the TERT promoter region; other promoter variants were not evaluated. For IDH mutation analysis, tumor tissue areas were selected after paraffin embedding. DNA isolation and amplification were performed, followed by real-time PCR and post-PCR fluorescence melting curve analysis (FMCA). IDH1 and IDH2 hotspot mutations were identified using fluorescent probes specific for the IDH1 and IDH2 hotspot gene regions. IDH1 R132H and IDH2 R172M are the most frequently detected IDH1 mutation sites. IDH2 R172M sequence is the most frequently detected mutation site. These are the codons that are looked at in the evaluation. Ninety percent of IDH mutations occur at codon 132. MGMT DNA extraction from an area containing at least 50% tumor tissue for mutation is done. Sections from tissues fixed in heavy metal-containing fixatives as well as sections from decalcified specimens were deemed suitable for pathological examination. During tissue decalcification, PCR amplification of genomic DNA can be performed. It is known to significantly degrade DNA. On the tissue sample obtained, 95% or 100% ethanol is added, and samples are taken into PCR tubes. Bisulfite and the tissue samples are mixed in the pipette. Computer-based percentage of methylated reference (PMR) values were calculated using dedicated software. Samples with PMR values greater than 2 were considered methylation-positive, whereas samples with PMR values below 2 were considered methylation-negative. Chromosome 7 abnormalities were analyzed by FISH on paraffin-embedded tissue sections using chromosome 7-specific alpha satellite DNA probes. FISH signals were recorded, classified according to standard cytogenetic criteria, and statistically analyzed. Loss of chromosome 10 was assessed by FISH analysis using probes specific for chromosome 10 on DNA obtained from paraffin-embedded tumor tissue sections. CDKN2A (p16) homozygous deletion was detected by FISH analysis performed on paraffin-embedded tumor-containing tissue sections using appropriate locus-specific probes. EGFR amplification was evaluated by FISH analysis on H&E-stained sections obtained from paraffin-embedded tumor tissue. 

Statistical analysis

The study's findings were evaluated and statistically analyzed using IBM SPSS Statistics for Windows, Version 22.0 (IBM Corp., Armonk, New York, United States). The suitability of parameters to normal distribution was evaluated using the Shapiro-Wilk test. Besides, definitive statistical parameters such as average, standard deviation, and frequency were evaluated. Pearson's chi-squared test was used to determine the groups causing a difference in the quantitative comparison of the findings. Pearson's correlation analysis was used to examine the correlation of parameters showing suitability to normal distribution. A value of p<0.05 was considered statistically significant for the study findings.

## Results

**Figure 1:**
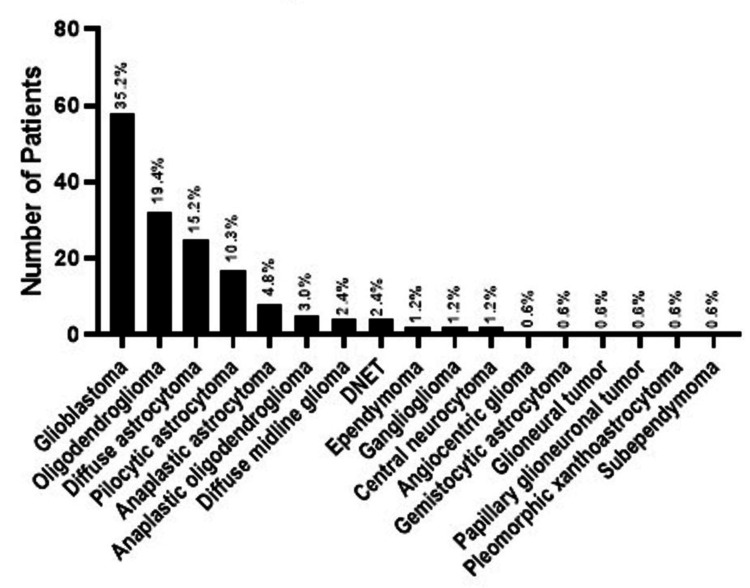
Diagnosis distribution of the patients DNET: dysembryoplastic neuroepithelial tumor

The mean age of the patients was 30.20±16.85 years, and there were no significant differences in age between the two genders. Most patients were diagnosed with glioblastoma, followed by oligodendroglioma, diffuse astrocytoma, and pilocytic astrocytoma (Figure 1).

**Figure 2:**
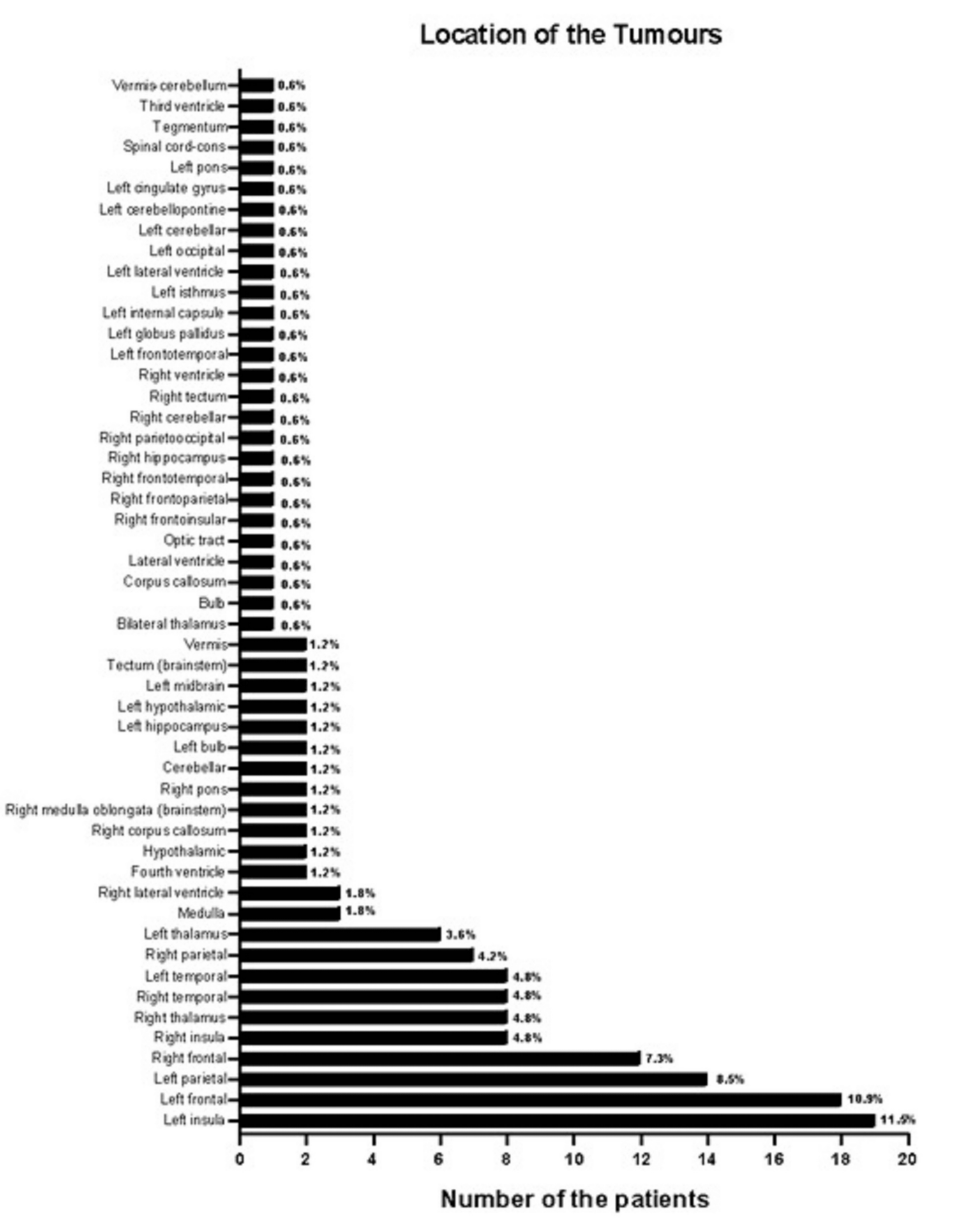
Tumor location distribution of the patients

**Figure 3:**
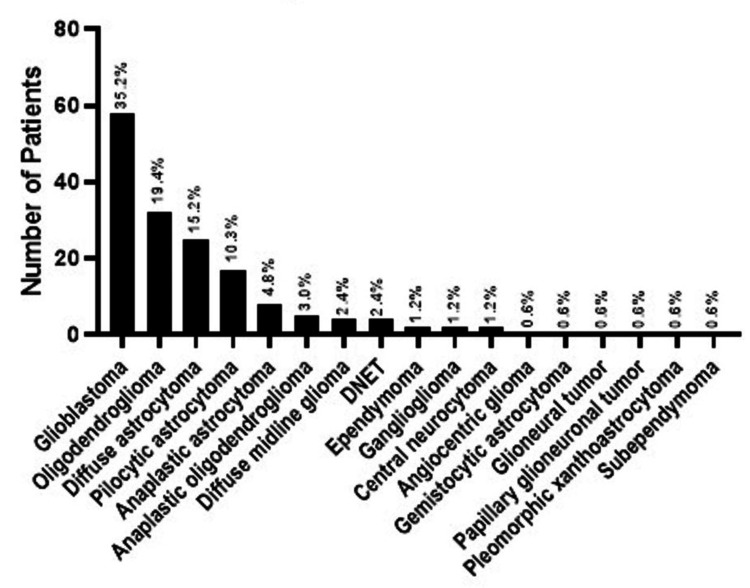
Relation of P53 and Ki67 positivity DNET: dysembryoplastic neuroepithelial tumor

**Figure 4:**
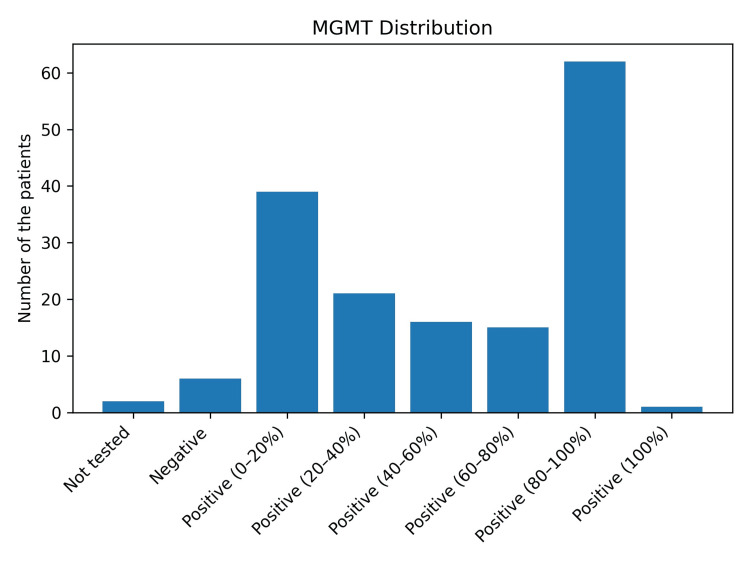
MGMT positivity distribution MGMT: O6-methylguanine-DNA methyltransferase

The tumors of the patients were primarily localized in the left insula and left frontal regions, followed by the left parietal and right frontal regions (Figure 2). A moderate and statistically significant correlation was observed between Ki-67 positivity and P53 positivity (Pearson's r=0.541; p<0.0001; Figure 3). Patients with grade 4 (36.97%) and grade 2 (36.36%) tumors were more prominent in the population, followed by grade 1 (17.58%) and grade 3 (9.09%). The positivity of the biopsy samples for MGMT revealed that most were 80-100% positive (Figure 4). ATRX, IDHR132, GFAP, EGFR, OLIG2, H3K27M, CD34, SYN, NEUN, NFP, PDL1, WT1, PTERT, P16, YAP1, GAP1, and 1p19q co-deletion positivity were similar in both male and female patients. However, there was a significant association between VIMENTIN positivity and gender (p<0.05), and it was found that the odds of being VIMENTIN positive are approximately 0.399 times the odds of being VIMENTIN negative for males compared to females, suggesting a lower likelihood of VIMENTIN positivity among male patients relative to female patients.

The most common 1p19q co-deletion positivity was observed in glioblastoma (36.6%) and oligodendroglioma (29.7%), followed by diffuse astrocytoma (16.8%; Table 1). Oligodendroglioma had the highest relative increase in observed 1p19q positivity, followed by glioblastoma, which also showed relevance. In contrast, tumor types such as dysembryoplastic neuroepithelial tumor (DNET) and ependymoma presented fewer positive cases than those projected by statistical models, suggesting a lesser correlation with 1p19q positivity. There were no significant differences between the Ki67 and P53 positivity according to 1p19q co-deletion positivity. There were no associations between 1p19q co-deletion positivity and MGMT positivity (Pearson's chi-square=0.14; p=0.700). On the other hand, MGMT positivity in percentage and 1q19p co-deletion positivity were found to be significantly associated (Table 2). There were no associations between diagnosis and MGMT positivity (Pearson's chi-square=112.451; p=0.055). There were no associations between ATRX, H3K27M, CD34, SYN, NEUN, NFP, PD-L1, VIMENTIN, WT1, P16, YAP1, and GAP. On the other hand, an IDHR132 mutation was positively associated with a 1p19q co-deletion (p<0.001; Table 3). Moreover, the odds ratio showed that GFAP positivity was associated with the positivity of 1p19q co-deletion (p=0.027; Table 3). Similarly, EGFR positivity was related to the positivity of 1p19q co-deletion (p=0.014; Table 3). Additionally, OLIG2 positivity was associated with the positivity of 1p19q co-deletion (p=0.002; Table 3).

**Table 1:** 1p19q positivity according to the diagnosis DNET: dysembryoplastic neuroepithelial tumor

Tumor type	Negative (%)	Positive (%)	Pearson's chi-square (p-value)
Anaplastic astrocytoma	1 (2.6)	5 (5)	62.234 (<0.0001)
Anaplastic oligodendroglioma	0 (0)	5 (5)	
Angiocentric glioma	1 (2.6)	0 (0)	
Central neurocytoma	1 (2.6)	1 (1)	
Diffuse astrocytoma	4 (10.5)	17 (16.8)	
Diffuse midline glioma	2 (5.3)	2 (2)	
DNET	4 (10.5)	0 (0)	
Ependymoma	1 (2.6)	0 (0)	
Ganglioglioma	2 (5.3)	0 (0)	
Gemistocytic astrocytoma	1 (2.6)	0 (0)	
Glioblastoma	9 (23.7)	37 (36.6)	
Oligodendroglioma	0 (0)	30 (29.7)	
Papillary glioneuronal tumor	0 (0)	1 (1)	
Pleomorphic xanthoastrocytoma	1 (2.6)	0 (0)	
Pilocytic astrocytoma	10 (26.3)	3 (3)	
Subependymoma	1 (2.6)	0 (0)	
Total negative vs. positive	38 (27)	101 (73)	-

**Table 2:** Association between MGMT positivity and 1p19q co-deletion positivity MGMT: O6-methylguanine-DNA methyltransferase

MGMT positivity	Negative (%)	Positive (%)	Pearson's chi-square (p-value)
Negative	1 (2.6)	4 (4)	16.585 (p=0.011)
Positive (0-20%)	6 (15.8)	27 (27)	
Positive (21-40%)	1 (2.6)	16 (16)	
Positive (41-60%)	2 (5.3)	11 (11)	
Positive (61-80%)	3 (7.9)	11 (11)	
Positive (81-99%)	24 (63.2)	31 (31)	
Positive (100%)	1 (2.6)	0 (0)	

**Table 3:** Associations between immunohistopathology results and 1p19q co-deletion positivity

Immunohistopathology results	Positive/negative	Negative (%) 1p19q	Positive (%) 1p19q	Pearson's chi-square (p-value)
ATRX	Negative (n (%))	12 (31.6)	27 (26.7)	0.321 (0.571)
	Positive (n (%))	26 (68.4)	74 (68.4)	
IDHR132	Negative (n (%))	32 (84.2)	49 (48.5)	14.469 (<0.001) (OR=46.22)
	Positive (n (%))	6 (15.8)	52 (51.5)	
GFAP	Negative (n (%))	7 (18.4)	6 (6.1)	4.8850 (0.027)
	Positive (n (%))	31 (81.6)	93 (93.9)	
EGFR	Negative (n (%))	21 (55.3)	32 (32.3)	6.092 (0.014)
	Positive (n (%))	17 (44.7)	67 (67.7)	
OLG2	Negative (n (%))	8 (21.1)	4 (4)	9.944 (0.002)
	Positive (n (%))	30 (78.9)	95 (96)	
H3K27M	Negative (n (%))	34 (89.5)	94 (94.9)	1.341 (0.247)
	Positive (n (%))	4 (10.5)	5 (5.1)	
CD34	Negative (n (%))	1 (2.6)	5 (5.1)	0.384 (0.536)
	Positive (n (%))	37 (97.4)	94 (94.9)	
SYN	Negative (n (%))	20 (52.6)	48 (48.5)	0.189 (0.664)
	Positive (n (%))	18 (47.4)	51 (51.5)	
NEUN	Negative (n (%))	27 (71.1)	59 (59.6)	0.106 (0.745)
	Positive (n (%))	11 (28.9)	40 (40.4)	
NFP	Negative (n (%))	19 (50)	43 (44.3)	0.353 (0.552)
	Positive (n (%))	19 (50)	54 (55.7)	
PD-L1	Negative (n (%))	18 (72)	54 (84.4)	1.708 (0.182)
	Positive (n (%))	7 (28)	10 (15.6)	
VIMENTIN	Negative (n (%))	3 (7.8)	16 (16.2)	1.571 (0.210)
	Positive (n (%))	35 (92.1)	83 (83.8)	
WT1	Negative (n (%))	37 (97.4)	96 (97)	0.015 (0.901)
	Positive (n (%))	1 (2.6)	3 (3)	
PTERT	Negative (n (%))	17 (73.9)	48 (90.6)	3.593 (0.058)
	Positive (n (%))	6 (26.1)	5 (9.4)	
P16	Negative (n (%))	7 (30.4)	19 (36.5)	0.528 (0.463)
	Positive (n (%))	16 (69.6)	33 (63.5)	
YAP1	Negative (n (%))	4 (26.7)	17 (48.8)	2.068 (0.150)
	Positive (n (%))	11 (73.3)	18 (51.4)	
GAB1	Negative (n (%))	3 (37.5)	5 (20.8)	0.889 (0.346)
	Positive (n (%))	5 (62.5)	19 (79.2)	

ATRX positivity was observed in 74 cases with 1p19q co-deletion positivity. Among these, 29 cases (39.2%) were diagnosed with oligodendroglioma, 25 cases (33.8%) were diagnosed with glioblastoma, six cases (8.1%) were diagnosed with diffuse astrocytoma, five cases (6.7%) were diagnosed with anaplastic oligodendroglioma, three cases (4.1%) were diagnosed with anaplastic astrocytoma, three cases (4.1%) were diagnosed with pilocytic astrocytoma, and one case (1.4%) was diagnosed with diffuse midline glioma. Anaplastic astrocytoma and ganglioglioma showed an equal distribution between IDHR132-positive and IDHR132-negative cases, indicating a balanced frequency of this mutation in these tumor types. Conversely, anaplastic oligodendroglioma, gemistocytic astrocytoma, and oligodendroglioma display a strong inclination towards IDHR132 positivity, with rates of 100%, 100%, and 96.8%, respectively, suggesting a significant association between these tumor types and the IDHR132 mutation. Diffuse astrocytoma presented a higher rate of IDHR132 positivity (64%), pointing to a prevalent mutation trend. In stark contrast, glioblastoma showed a predominant IDHR132 negativity (86%), with only a tiny fraction being positive (14%), indicating that IDH mutations are less common in this aggressive tumor type. There were no significant differences between the Ki67 and P53 positivity according to 1p19q co-deletion positivity. Anaplastic astrocytoma, anaplastic oligodendroglioma, and angiocentric glioma demonstrated high GFAP positivity, with rates at 87.5%, 100%, and 100%, respectively, reflecting a strong astrocytic component. Diffuse astrocytoma and diffuse midline glioma showed high GFAP positivity rates at 96% and 100%, respectively. Despite being highly aggressive, glioblastoma maintained a substantial astrocytic presence, as evidenced by an 87.7% GFAP positivity rate. Oligodendroglioma, typically known for its oligodendrocytic nature, surprisingly showed a high GFAP positivity at 96.8%. Anaplastic astrocytoma and anaplastic oligodendroglioma showed substantial EGFR positivity at 87.5% and 80%, respectively. The data for diffuse astrocytoma and ganglioglioma showed a balanced EGFR positivity rate, approximately 50%, indicating variability within these tumors. Meanwhile, diffuse midline glioma and ependymoma exhibited higher EGFR positivity at 75% and 100%, respectively. Glioblastoma, a highly aggressive tumor type, presented a high rate of EGFR positivity at 70.2%. Oligodendroglioma and pleomorphic xanthoastrocytoma demonstrated considerable EGFR positivity at 77.4% and 100%. Anaplastic astrocytoma, anaplastic oligodendroglioma, diffuse midline glioma, ganglioglioma, gemistocytic astrocytoma, glioneuronal tumor, oligodendroglioma, papillary glioneuronal tumor, and pleomorphic xanthoastrocytoma all showed a 100% positivity rate for OLIG2. In contrast, angiocentric glioma, ependymoma, and subependymoma exhibited 100% OLIG2 negativity. Glioblastoma, a notably aggressive tumor type, displayed substantial OLIG2 positivity (87.7%), indicating that despite its aggressive nature, it retains or mimics characteristics of oligodendroglial lineage cells. While predominantly negative for H3K27M, glioblastoma and pilocytic astrocytoma showed a small percentage of cases with the mutation (7% and 5.9%, respectively). Diffuse midline glioma showed a 100% positivity rate for the H3K27M mutation.

The data revealed complete CD34 positivity (100%) in several tumor types including anaplastic astrocytoma, angiocentric glioma, central neurocytoma, diffuse midline glioma, ependymoma, ganglioglioma, gemistocytic astrocytoma, glioblastoma, glioneuronal tumor, papillary glioneuronal tumor, pleomorphic xanthoastrocytoma, pilocytic astrocytoma, and subependymoma. On the other hand, diffuse astrocytoma and oligodendroglioma exhibited predominantly CD34-positive results with 88% and 93.5% positivity rates, respectively.

## Discussion

Gliomas are a group of tumors with different histological and molecular grades and varying clinical courses [9]. In adults, the incidence of gliomas varies between 1.9 and 9.6 per 100,000 cases depending on different factors, including age, gender, geographical region, and ethnicity. The 2016 WHO central nervous system classification emphasized molecular markers, thereby enhancing diagnostic accuracy, prognosis estimation, and the formulation of treatment protocols in glial tumors through molecular studies [2]. The latest classification of central nervous system tumors in 2021 placed greater importance on biomarkers for diagnosis [3]. In the present study, we investigated the relation between histopathological alterations and different types of central nervous system tumors. In our study, 162 patients were included in the analysis, with a mean age of 39.20±16.85 years. The patient cohort consisted of 93 males (56.4%) and 72 females (43.6%), indicating a slight predominance of male patients. On the other hand, the mean age for male patients was 37.70 years (SD=17.62), while the mean age for female patients was 41.14 years (SD=15.70). Statistical analysis revealed no significant difference in age between male and female patients, suggesting that age distribution is comparable across genders. The most common diagnosis was glioblastoma, accounting for 35.2% of our patient population. Glioblastoma is a highly aggressive and common type of primary brain tumor in adults, characterized by rapid growth and poor prognosis [10]. The high frequency observed in this cohort is consistent with epidemiological data indicating glioblastoma as the most prevalent malignant primary brain tumor [11]. Oligodendroglioma was the second most frequent diagnosis, representing 19.4% of cases. The substantial occurrence of oligodendroglioma in this patient group underscores its clinical significance and the importance of molecular diagnostics in guiding therapeutic strategies. In the relevant literature, oligodendrogliomas are the second most common intraparenchymal brain tumors in adults [1,2]. Our results revealed that the tumors were predominantly located in the left insula and left frontal regions, followed by the left parietal and right frontal regions. This distribution suggests a predilection for tumor development in the left hemisphere, particularly in areas associated with higher cognitive functions and motor control [12,13]. The left insula and frontal lobes are integral to a range of functions, including language, emotional processing, and executive functions, such as decision-making and problem-solving. The reason why insular tumors were more common in our study is that our center is a center where specific tumors are treated surgically. The association between tumor grade and 1p19q co-deletion positivity reveals significant insights into brain tumors' molecular characterization and clinical behavior. In this study, the distribution of 1p19q co-deletion varied markedly across tumor grades. The typically favorable prognosis associated with the 1p19q co-deletion in diffuse low-grade and anaplastic oligodendrogliomas is due to several factors [14]. This genetic alteration is associated with a better response to therapies, including chemotherapy and radiotherapy, which improve survival rates. However, the present study's finding that high-grade tumors, such as glioblastomas, also showed a significant prevalence of 1p19q co-deletion but did not display the same favorable outcomes raises essential questions. One reason for this situation may be tumor heterogeneity. High-grade tumors, including those with the 1p19q co-deletion, exhibit greater genetic and molecular heterogeneity compared to lower-grade tumors [15-17]. This complexity can lead to varied and less predictable treatment responses, diminishing the co-deletion's favorable impact on prognosis. Secondly, high-grade tumors such as glioblastomas are inherently more aggressive and have a more invasive growth pattern [18]. This aggressive behavior can overshadow the potential benefits of the 1p19q co-deletion, leading to poorer overall survival despite genetic alteration. Moreover, 1p19q co-deletion may play different roles in the pathogenesis and progression of high-grade tumors compared to low-grade tumors. In high-grade tumors, additional genetic alterations and molecular pathways that drive tumor aggressiveness and resistance to treatment might counteract the positive effects typically associated with the co-deletion [19]. The MGMT gene plays a crucial role in tumor resistance to alkylating agents used in chemotherapy, such as temozolomide, commonly employed in treating glioblastoma and other high-grade gliomas [19-21]. MGMT works by removing alkyl groups attached to the O6 position of guanine, a modification induced by alkylating agents [20]. The methylation status of the MGMT promoter is a pivotal biomarker in neuro-oncology because it inversely correlates with the expression of the MGMT enzyme: hypermethylation leads to gene silencing and increased sensitivity to alkylating chemotherapy, while an unmethylated status is associated with therapeutic resistance [22].

The association between MGMT promoter methylation and 1p19q co-deletion positivity in brain tumors reveals critical insights into the genetic and epigenetic interactions that influence tumor behavior and treatment response. Our study showed that while only 4% of tumors with no MGMT methylation showed 1p19q co-deletion positivity, this rose significantly among tumors with varying degrees of MGMT methylation, indicating a clear correlation between these two markers (chi-square=16.585; p=0.011).

Specifically, tumors with low (0-20%) and moderate (21-60%) levels of MGMT methylation showed higher rates of 1p19q co-deletion positivity, suggesting that even partial methylation of the MGMT promoter can contribute to the favorable outcomes typically seen in tumors with this genetic alteration. This finding is consistent with Hegi et al. [23], who demonstrated that MGMT methylation leads to enhanced sensitivity to alkylating agents, thereby improving survival rates in patients with glioblastoma. Furthermore, the association of high levels of MGMT methylation (61-80%) with 1p19q co-deletion positivity suggests a synergistic effect that enhances therapeutic efficacy, as observed in studies where MGMT methylation combined with 1p19q co-deletion resulted in better treatment responses and longer progression-free survival. However, an interesting observation was that tumors with very high (81-99%) and complete (100%) MGMT methylation had lower rates of 1p19q co-deletion positivity, which might indicate that extremely high levels of MGMT methylation could mitigate the additive benefits of the 1p19q co-deletion [24,25]. This nuanced relationship underscores the complexity of tumor biology, where both genetic and epigenetic factors interplay to influence tumor behavior and response to therapy [26]. In our study, MGMT positivity across different brain tumor types gives a variable expression of the MGMT profile, which could potentially influence treatment strategies and prognostic outlooks for patients with these tumors. Anaplastic astrocytoma is characterized by increased cellular atypia and mitotic activity compared to its lower-grade counterparts. In our study, we observed a spread across MGMT positivity, with a notable representation in the lower ranges (0-20%, 21-40%), while small proportions were found in higher MGMT positivity ranges (81-99%). The presence of MGMT in lower ranges suggests that a subset of these tumors may have methylated MGMT promoters, leading to the reduced expression of the MGMT protein and potentially increased sensitivity to alkylating agents. This feature might benefit clinical settings where temozolomide is considered part of the standard care regimen, potentially leading to better responses and extended survival. There were no associations between ATRX, H3K27M, CD34, SYN, NEUN, NFP, PD-L1, VIMENTIN, WT1, P16, YAP1, and GAP. The presence of an IDHR132 mutation, GFAP positivity, EGFR positivity, and OLIG2 positivity was positively associated with a 1p19q co-deletion. When we examined the relationship between 1p19q and other biomarkers, we observed a correlation with biomarkers frequently detected in oligodendrogliomas.

Our study also had some limitations. One of the primary limitations of this study is the relatively small and potentially non-representative sample size. Although the study provides valuable insights into the molecular and clinical characteristics of various brain tumors, the limited number of cases for certain tumor types, such as anaplastic oligodendrogliomas and central neurocytomas, restricts the generalizability of the findings. The lack of diversity in the patient population may also affect the applicability of the results to broader and more varied populations, which could exhibit different molecular profiles and clinical outcomes. Our study was planned in a single institution. This single-center approach limits the ability to generalize the findings to other settings where diagnostic criteria, treatment protocols, and patient demographics may differ. Our study has limits due to its cross-sectional design, which captures a snapshot in time but does not account for changes in tumor characteristics, patient health, or treatment efficacy over time. Lastly, the study focused on genetic markers and their correlation with tumor types. Still, it did not investigate the functional impact of these mutations on tumor behavior or treatment response. Understanding the functional consequences of genetic alterations is crucial for developing targeted therapies and predicting patient outcomes. The lack of functional data limits the ability to conclude the mechanisms driving tumor progression and response to therapy. The most significant finding in our study was the interesting relationship between 1p19q and MGMT. While 1p19q correlated with certain amounts of methylation of MGMT, there was no correlation with 1p19q when it was high and completely methylated. This finding led us to think that high and complete MGMT methylation levels may reduce the beneficial activity of 1p19q co-deletion. While our study provides a broad perspective about the histopathological markers in the diagnosis of various brain tumors, it highlights the need for larger, multi-center studies with comprehensive molecular analyses and longitudinal follow-up to better understand the complex landscape of brain tumor biology and improve patient clinical outcomes.

## Conclusions

Although 1p19q co-deletion is a defining diagnostic feature of oligodendroglioma, it was also detected in a substantial proportion of other glial and glioneuronal tumors in our cohort, most notably glioblastoma. The presence of 1p19q co-deletion in non-oligodendroglial tumors, together with its observed association with MGMT promoter methylation, highlights the biological complexity of central nervous system tumors and suggests a multifaceted interplay between genetic and epigenetic factors influencing tumor behavior and therapeutic response.

These findings should be interpreted in the context of the study's retrospective and cross-sectional design. The relatively limited cohort size and inclusion of heterogeneous glial tumor subtypes restrict the strength of biologically specific and causal inferences, particularly regarding the role of 1p19q alterations beyond canonical oligodendroglial tumors. Accordingly, this study should be regarded as a hypothesis-generating, exploratory investigation rather than one providing definitive mechanistic conclusions.

Additional limitations include the single-center setting, heterogeneity of tumor entities, and the relatively broad operational definition of 1p19q positivity. Moreover, the lack of entity-stratified and multivariable analyses adjusting for major confounders, such as tumor type, tumor grade, and IDH mutation status, may have influenced some observed associations. These considerations should be taken into account when interpreting the results. Future prospectively designed studies with larger, more homogeneous, and potentially multi-center cohorts, using standardized molecular methodologies and stratified analytical approaches, are warranted to validate and extend these observations.
